# The Roles of Various Immune Cell Populations in Immune Response against Helminths

**DOI:** 10.3390/ijms25010420

**Published:** 2023-12-28

**Authors:** Janina Lekki-Jóźwiak, Piotr Bąska

**Affiliations:** 1 Division of Parasitology and Parasitic Diseases, Department of Preclinical Sciences, Institute of Veterinary Medicine, Warsaw University of Life Sciences, 02-786 Warsaw, Poland; janina_lekki-jozwiak@sggw.edu.pl; 2 Division of Pharmacology and Toxicology, Department of Preclinical Sciences, Institute of Veterinary Medicine, Warsaw University of Life Sciences, 02-786 Warsaw, Poland

**Keywords:** helminths, immune response, macrophages, basophils, eosinophils, neutrophils, ILC2, T cells, B cells, ETC, granuloma

## Abstract

Helminths are multicellular parasites that are a substantial problem for both human and veterinary medicine. According to estimates, 1.5 billion people suffer from their infection, resulting in decreased life quality and burdens for healthcare systems. On the other hand, these infections may alleviate autoimmune diseases and allergy symptoms. The immune system is programmed to combat infections; nevertheless, its effector mechanisms may result in immunopathologies and exacerbate clinical symptoms. This review summarizes the role of the immune response against worms, with an emphasis on the Th_2_ response, which is a hallmark of helminth infections. We characterize non-immune cells (enteric tuft cells—ETCs) responsible for detecting parasites, as well as the role of hematopoietic-derived cells (macrophages, basophils, eosinophils, neutrophils, innate lymphoid cells group 2—ILC2s, mast cells, T cells, and B cells) in initiating and sustaining the immune response, as well as the functions they play in granulomas. The aim of this paper is to review the existing knowledge regarding the immune response against helminths, to attempt to decipher the interactions between cells engaged in the response, and to indicate the gaps in the current knowledge.

## 1. Introduction

The word “helminth” comes from the Greek term for “worm”. The three primary categories of helminths that infect humans are nematodes (roundworms), acanthocephalans (thorny-headed worms), and platyhelminths (flatworms) [1,2]. Helminths have affected humans since ancient times, as indicated by the finding of calcified helminth eggs in mummies dating back to 1200 B.C. Helminths also played a role in the Cold War during the Chinese invasion of Taiwan (formerly Formosa), when thousands of Chinese soldiers were required to swim to reach the island’s shore. After swimming in Chinese canals, these soldiers developed symptoms such as fever, abdominal pain, and skin rashes. The investigation revealed that these symptoms were caused by *Schistosoma japonicum* [3,4,5].

Helminths are a divergent group of parasites that pose a global burden to public health. Soil-transmitted helminths (STH), such as hookworm, *Ascaris,* and whipworm, are among the most widespread infections globally, impacting nearly 1.5 billion individuals [6]. Additionally, Filariodea nematodes and *Schistosoma* spp. flukes affect over 120 million [7] and 240 million people [8], respectively, with millions more at risk. Controlling these diseases is challenging due to the various niches occupied in the host and their divergent life cycles. STH parasites inhabit the intestine, and their eggs are released into the environment through feces. Subsequent ingestion of embryonated eggs or infectious larvae by another individual perpetuates the life cycle.

Filarial nematodes release larvae into the bloodstream, which are ingested by mosquitoes and transmitted to another host following development in the mosquito’s tissues. *Schistosoma* spp. eggs are excreted in stool or urine and develop into miracidium, which infects a snail and undergoes further development, resulting in the release of infectious stages known as cercariae that enter the final host. These infections are particularly prevalent in regions with endemic, tropical conditions, primarily in developing countries that struggle with inadequate access to proper sanitation, clean water, and low hygiene and healthcare standards [9,10]. Nevertheless, due to climate change, helminth and other infectious diseases associated with tropical and subtropical regions are becoming burdens for other regions [11,12].

While current chemotherapy is effective and widely administered, it does not result in long-term protection against the high rates of reinfection. It is estimated that approximately 50% of patients treated with these drugs will experience reinfection within six months upon their initial treatment [13]. To address concerns about overusing drugs and the development of drug resistance in parasite populations, the World Health Organization (WHO) places significant emphasis on the development of effective vaccines. Unfortunately, currently, there is no efficient vaccine against helminths, despite numerous research efforts [14,15,16,17,18,19]. Nevertheless, several potential vaccines have been identified and have advanced to the second phase of clinical trials, offering hope for their introduction to the market, which could reduce the reliance on anthelmintic drugs [20].

For many years, helminth infections have been overlooked, leading to their classification by the Centre for Disease Control and Prevention (CDC) as NPI—neglected parasitic infections. Presently, knowledge regarding helminth infections is being thoroughly examined, yet certain gaps in our understanding still persist. Gaining insight into the interaction between helminths and the immune system will facilitate more efficient vaccine design and better control of the infections.

On the other hand, while helminths have a negative impact, they may also be beneficial for humans by alleviating the symptoms of diet-induced insulin resistance [21], colitis [22], Crohn’s disease [23], and reducing allergic symptoms through an IL-10-dependent mechanism [24]. This review provides a concise overview of the mechanisms involved in the recognition of helminths by host cells and the roles of various cell types, including macrophages, tuft cells, T and B lymphocytes, eosinophils, basophils, neutrophils, innate lymphoid cells type 2 (ILC2), and mast cells.

## 2. The Immune Response to Helminth Infection

During helminth infections, parasites can cause significant tissue damage and release excretory–secretory (ES) products that trigger a type 2 immune response. This response is characterized by the recruitment and activation of innate immune cells, including dendritic cells, macrophages, mast cells, ILC2s, basophils, and eosinophils, with a hallmark of increased IL-4, IL-5, IL-9, and IL-13 release, leading to Th_2_ and B cells activation and the production of IgE and IgG1 class antibodies. Collectively, these molecular and cellular events initiate host-protective responses, including mucus production by goblet cells, proliferation of epithelial cells, and smooth muscle contractions, ultimately resulting in helminth expulsion.

## 3. Detection

### 3.1. PRRs in the Recognition of Helminths

Detecting the presence of an invading pathogen represents a crucial initial step in initiating a specific and effective immune response. The immune system identifies infections or tissue damage through pattern recognition receptors (PRRs), which fall into three main classes: toll-like receptors (TLRs), C-type lectin receptors (CLRs), and NOD-like receptors (NLRs).

**Figure 1:**
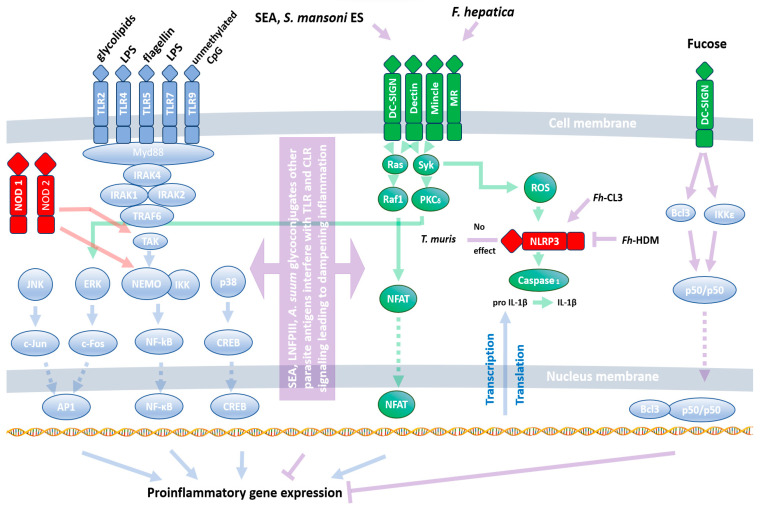
Interaction of helminth antigens with PRRs. Natural ligands of PRRs (TLRs, CLRs, and NLRs) induce a proinflammatory response through the activation of the transcription factors NF-κB, AP-1, CREB, and NFAT. Blue, green, and red arrows represent classical signaling from TLRs, CLRs, and NLRs, respectively. Violet arrows indicate interference of helminths with PRR signaling. The parasites stimulate PRRs directly or interfere with their signaling pathways, inducing a Th_2_/Th_reg_ response. A full description is given in the text (Section 3.1). *Fh*-CL3—F. hepatica cathepsin L3, *Fh*-HDM—fasciola hepatica helminth defense molecule, SEA—schistosoma-soluble egg antigens.

Pattern recognition receptors (PRRs) are responsible for recognizing pathogen-associated molecular patterns (PAMPs) and danger-associated molecular patterns (DAMPs). Examples of PAMPs include LPS, dsRNA, and lipomannan/lipoarabinomannan, while DAMPs encompass extracellular ATP, HSPs, and mtDNA, among others. This intricate system allows the immune system to distinguish between different invading threats and mount an appropriate response. Both TLRs and CLRs are cell membrane-anchored proteins; however, their signaling pathways are different (Figure 1). TLRs (except TLR3) activate NF-κB through Myd88/IRAKs/TRAF activation [25], while CLRs are distinguished in two classes: those containing the immunoreceptor tyrosine-based activation motif (ITAM) signal, propagating through spleen tyrosine kinase (Syk), and those containing the immunoreceptor tyrosine-based inhibitory motif (ITIM), propagating through Src homology region 2 domain-containing phosphatase-1 (Shp-1) [26]. NLRs are intracellular receptors that induce proinflammatory signals or inflammasome formation, leading to NF-κB, IL-1β, and IL18 precursor activation [27]. A more detailed description of PRRs and theirs ligands and signaling is beyond the scope of this review and may be found in other reviews [26,28,29].

While our understanding of bacterial and viral PAMPs and their corresponding PRRs is well-established, our knowledge regarding their ability to recognize helminths is still in its infancy. Despite this, all three classes of PRRs have been demonstrated to play a role in the response to helminth infections. Typically, PRRs induce a proinflammatory response. However, their function during helminth infections differs from their response to other pathogens. Instead of recognizing evolutionarily conserved worm antigens, PRRs are rather utilized by the invaders as tools to facilitate infection. This unique interaction is beneficial for both the host and the parasite. The parasite can release eggs, and the host is spared from the potential harm caused by prolonged inflammation. B cells from filarial-infected humans stimulated with TLR2, TLR4, and TLR9 ligands (Pam3Cys, LPS, and CpG, respectively) show diminished expression of activation markers and IL-6, IL-10, and TNF-α [30]. Moreover, Pam3Cys and HKLM (TLR2 ligands) do not affect IFN-γ, TNF-α, IL-12, IL-1β, IL-6, IL-17, or IL-23 release among infected patients’ PBMCs, but they do upregulate their expression among patients showing chronic immunopathology associated with enhanced phosphorylation of p38 MAPK and ERK1/2 [31]. Another filarial nematode, *Acanthocheilonema viteae,* releases ES-62 antigen, which dampens TLR4 signaling, making cells refractory to its ligands [32]. Shifting PRR signaling from Th_1_ towards Th_2_ is also observed in *Schistosoma japonicum*-infected mice, which showed elevated expression of TLR2, TLR3, TLR4, and TLR7 in the lungs. However, stimulation with TLRs’ cognate ligands results in a substantially higher release of IL-4 than IFN-γ [33].

The main component interfering with TLR4 signaling seems to be *S. mansoni* egg carbohydrate, lacto-N-fucopentaose III (LNFPIII) activating extracellular signal-regulated kinase (ERK) [34]. Moreover, Schistosoma-soluble egg antigens (SEA) and *S. mansoni* E/S are bound by DC-SIGN, MGL, MR, and MR, respectively, leading to an inhibition of DC activation by TLR ligands [35]. This results in a reduction in LPS-induced release of IL-10, IL12p40, TNF-α, IL12p70, and IL-6 [33] and a diminishment of the proinflammatory response [36]. The fluke *Fasciola hepatica* favors the production of IL-10 and IL-27p28 by LPS-stimulated DC through DC-SIGN interactions [37]. Similarly, *Ascaris sum* glycoconjugates diminish CD40, CD80, CD86, and MHCII expression induced by LPS through CLRs: DC-SIGN and MR [38]. DC-SIGN signaling counteracting the Th_1_ response is likely to be associated with parasite-derived fucose, which induces IKKε and CYCLD-dependent Bcl and NF-κB p50/p50 dimer activation [39,40].

Both TLRs and CLRs were also shown to have a similar effect during *Nippostrongylus brasiliensis* infection. TLR4 induces a Th_2_ response through IFN type I induction [41], while SP-D activation results in strong IL-4 and IL-13 release [42]. On the other hand, infection with *Heligmosomoides polygyrus* shows no impact of TLR2, TLR4, TLR5, and TLR9 on egg output [43]. This suggests other redundant mechanisms during infection; for example, through the reduction of DC Dectin-1 expression that drives Th_1_/Th_17_ development [44]. The above-mentioned effects are associated with the development of the Th_2_ response through the utilization of TLRs and CLRs by the worms. On the other hand, the NLRP3 inflammasome seems to be resistant to parasites’ modulation and induces a proinflammatory response against *Trichuris muris* infection [45]. Nevertheless, helminths’ molecules may have a pivotal impact on NLRP3 activation: *F. hepatica* cathepsin L3 (*Fh*-CL3) induces its activation, whereas *F. hepatica* helminth defense molecule (*Fh*-HDM) prevents it [46].

### 3.2. Detection of Helminths by Intestinal Epithelial Cells (IEC)

Another critical type of cell engaged in helminth detection is the intestinal epithelial cells (IECs), which serve as the primary physical barrier for helminths in the gastrointestinal tract. IECs encompass various cell types, including enterocytes, enteric tuft cells (ETCs), M cells, and enteroendocrine cells (EECs), and they release DAMPs or alarmins that are involved in helminth detection. DAMPs are released in response to cellular damage or when ligands bind to toll-like receptors (TLR). Damaged IECs release cytokines such as IL-33, IL-25, and thymic stromal lymphopoietin (TSLP) [13], playing a crucial role in worm infections. Mice lacking any of these cytokines exhibit reduced Th_2_ immune responses. The most powerful population of IEC engaged in worms recognition are ETCs. They release IL-25 and express taste 2 receptors (TAS2Rs), which typically recognize bitter tastes in taste cells on the tongue. However, within the intestinal epithelium, they serve as parasite sensors. This has been demonstrated using an inhibitor of bitter taste receptors, allyl isothiocyanate, which, upon delivery to intestinal villi, reduces IL-25 release from tuft cells during *Trichinella spiralis* infection [47].

M cells, constituting approximately 10% of IECs during homeostasis, serve as a link between the intestinal lumen and the immune system. They possess reduced microvilli, and unlike other IECs, specialize in capturing antigens and delivering them to lymphocytes or macrophages [48]. Studies have shown that there is no change in the function or number of M cells during *H. polygyrus* infection in mice [49]. Nevertheless, further research is required to ascertain the extent of M cells’ involvement in the immune response against other helminths.

Another category of intestinal epithelial cells (IECs) that is likely to play a role in pathogen recognition is enteroendocrine cells (EECs). The principal PRRs found on EECs include TLR4, TLR5, and TLR9 [50,51,52]. These cells can release not only peptide hormones but also cytokines in response to molecules associated with pathogens [53]. It has been shown that EECs downregulate cholecystokinin (CCK) production during *N. brasiliensis* in rats [54]. On the other hand, mice infected with *T. spiralis* exhibit hyperplasia and heightened synthesis of CCK-secreting cells, leading to an increase in serum CCK levels [55]. This phenomenon leads to the conclusion that alterations in cholecystokinin (CCK) levels appear to be species- and host-dependent. CCK triggers muscle contractions in the small intestine [56] and induces hypophagia [57]. The reinforcement of contractions facilitates the expulsion of parasites, while hypophagia allows the host to prioritize an immune response over the digestive process. Moreover, parasite species may not only have a pivotal impact on host cells, but cells’ responses to a parasite isolate may also differ [58,59].

## 4. Type 2 Inflammatory Cells—Development and Function

### 4.1. Enteric Tuft Cells (ETC)

The previous section summarized the role of ETCs in helminth recognition. This paragraph will focus on their general description and interplay with hematopoietic cells. Since 2016, special attention has been given to the role of intestinal tuft cells (ETC) in parasitic infections [60,61]. Under normal physiological conditions, ETCs in adult mice make up approximately 0.5% of the population of intestinal epithelial cells (IEC), but this number increases during helminth infections. Infection of mice with *N. brasiliensis* results in a tenfold increase in the number of ETCs [62]. ETCs originate from stem cells in intestinal crypts, and their differentiation depends on POU domain class 2 transcription factor 3 (POU2F3) and growth factor independent 1b (Gfi1b) [60]. ETCs can be divided into two subtypes: ETC1 (neuronal) and ETC2 (immune). ETC1 cells predominate under homeostasis, while ETC2 cells are more prevalent during parasitic infections [63]. Interestingly, the development of both ETC1 and ETC2 is significantly regulated by neurons, which was proven using three-dimensional organoid cultures. Cultures deprived of neurons or pilocarpine supplementation (a cholinergic agonist) showed a reduction in the number of ETCs within a week [64]. This emphasizes the interplay between the immune and nervous system during infection, as described previously [65].

**Figure 2:**
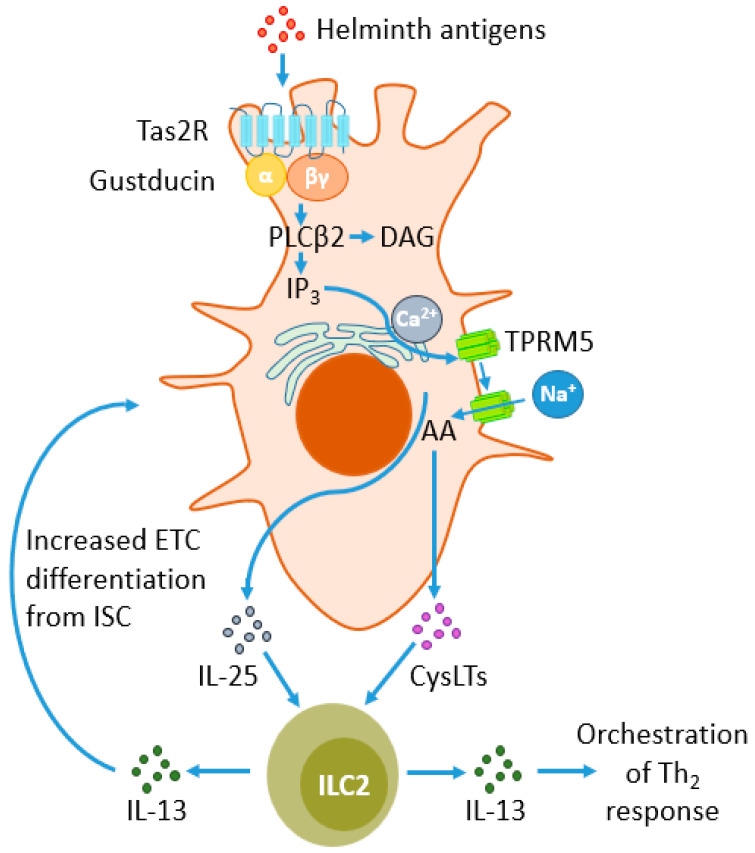
Response of enteric tuft cells (ETCs) to parasite antigens and their impact on type 2 innate lymphoid cells (ILC2s). Type 2 taste receptors (TAS2Rs) belong to G protein-coupled receptors (GPCRs) and sense parasites’ antigens, signaling through the G protein gustducin. Gustducin activates phospholipase β2 (PLCβ2), which hydrolyzes phosphatidylinositol biphosphate (PIP2) to diacylglycerol (DAG) and inositol trisphosphate (IP3). The latter compound induces the migration of calcium from the endoplasmic reticulum (ER) towards the cytoplasm, facilitating the influx of sodium ions into the cell through transient receptor potential cation channel subfamily M member 5 (TRPM5). This leads to the metabolism of arachidonic acid (AA) to cysteine leukotrienes (CysLTs) and the release of CysLTs and IL-25. Both mediators stimulate ILC2s to release IL-13, a powerful modulator of the Th_2_ response. There is also a positive feedback loop between the secretion of IL-25 and CysLT from ETC. ILC2s, through the release of IL-13, stimulate intestinal stem cells (ISC) to differentiate into ETC, increasing their number during helminth infections.

ETCs also serve as the primary source of IL-25 and generate cysteinyl leukotrienes (CysLT), which activate ILC2s to produce IL-13 [66] (Figure 2) and control further events during the immune response. IL-25 assumes a significant role in coordinating the intestinal response to helminth infections, a fact underscored by its multifaceted impact on various influencing factors. It triggers the production of Th_2_ cytokines such as IL-4, IL-5, and IL-13 by activating ILC2s, and it fosters the activation and differentiation of CD4^+^ T cells into Th_2_ cells. Furthermore, IL-25 induces the contraction of intestinal smooth muscle cells, facilitating the expulsion of worms during nematode infection [67]. Beyond its role in promoting type 2 responses, research indicates that it has the capability to suppress Th_1_ and Th_17_ responses across diverse environments, including the intestine [68].

### 4.2. Macrophages (Mφ)

Human intestinal macrophages have an approximate lifespan of one year [69] and can be categorized into three subtypes: mature macrophages derived from monocytes (Mm), inflammatory macrophages derived from monocytes (Mi), and self-maintaining macrophages (Ms). All three subtypes are located in the lamina propria of the intestinal mucosa, just beneath the epithelial cells. Additionally, Ms are also present in the submucosal and outer muscular layers. Ms are distinguished by their unique ability to self-regenerate and maintain their phenotype without the need for external activating signals. Mm and Mi release anti-inflammatory and pro-inflammatory cytokines, respectively, while Ms actively engage in interactions with the enteric nervous system (ENS), relying on noradrenaline signaling through intestinal neurons to facilitate gut peristalsis [70].

During helminth infections, lamina propria macrophages produce various factors such as IL-4, IL-13, IL-10, arginase-1 (Arg-1), chitinase-like protein (Ym1), and resistin-like alpha [71]. Another classification of macrophages is based on their phenotype: classically activated (M1) and alternatively activated (M2) macrophages, with the latter playing a significant role in the development of a protective response during helminth infections. M2 activation primarily involves IL-4 and IL-13 triggering the common receptor IL-4-α (IL-4Rα) signaling. Typically, IL-4 and IL-13 originate from ILC2s, basophils, or IECs. Helminths have a vested interest in inhibiting a detrimental Th_1_ response, and they release additional factors that induce M2 differentiation [72]. Mouse M2 macrophages upregulate Arg1 production, leading to the synthesis of L-ornithine, which directly inhibits the movement of *H. polygyrus* larvae [73]. Notably, during *N. brasiliensis* infection, arginase-dependent smooth muscle contractions occur [74], enhancing physical parasite expulsion. Interestingly, the ability of human macrophages to express arginase remains a subject of debate [75]. Other powerful macrophage-derived orchestrators of immune response are Ym1 and resistin-like molecule alpha (RELMα). Ym1 acts as a chemoattractant for eosinophils [76] and also participates in cell–cell and cell–extracellular matrix (ECM) interactions through binding to heparin [77]. RELMα is thought to have a regulatory role in inhibiting excessive type 2 responses. Mice with a deficiency in the RELMα gene exhibit significant liver, intestinal, and lung pathology in *S. mansoni* and *N. brasiliensis* infection models, characterized by an excessive Th_2_ cytokine response, fibrosis, and inflammation [78].

Moreover, M macrophages cannot be seen only as cells engaged in combating the invader, as their role is much more complex. They are powerful drivers of tissue repair and remodeling during and after the infection. M2s promote higher levels of vascular endothelial growth factor (VEGF), insulin-like growth factor 1 (IGF-1), matrix metalloproteinases (MMP), platelet-derived growth factor (PDGF), and the angiogenic Fizz1 protein, which also stimulates actin and collagen synthesis [79]. IL-10 is a well-known anti-inflammatory cytokine that is closely associated with M2 responses in parasitic worm infections [80]. Therefore, macrophages in type 2 responses serve the three “R” functions: react, resolve, and repair.

### 4.3. Basophils

Basophils are a short-lived type of white blood cell, with a lifespan of 1–2 days [81]. They are characterized by the presence of basophilic granules in their cytoplasm. Under normal physiological conditions, they constitute approximately 0.5–1% of the total leukocyte count, but this percentage increases during the Th_2_ immune response. Interestingly, unlike in mice, humans rarely exhibit basophilia during parasitic infections [82]. Mature basophils circulate in the bloodstream and migrate to damaged tissues during inflammatory responses, and their numbers rise in response to IL-3, TSLP, IL-25, and IL-33 [83,84]. Basophils produce various cytokines, such as IL-4, IL-5, IL-13, TSLP, prostaglandins, leukotrienes (which can activate ILC2s), and IL-5 (which induces eosinophil recruitment) [85].

The release of IL-4 and IL-13 by basophils can be triggered by TLR2, although this signal often falls short of activating the pathways responsible for releasing leukotrienes or histamine [86]. Basophils also play a role in secondary immune responses, as they carry the FcεR1 receptor on their surface. Depleting basophils or removing FcεR1 from their surface results in reduced M2 polarization [87]. While basophils do express MHC-II, their classification as professional antigen-presenting cells (APCs) is a matter of debate due to their relatively low surface MHC-II levels. In vivo studies have revealed that basophil–T cell interactions in lymph nodes are typically brief and unstable [88].

Nevertheless, it is important to highlight that basophils do not consistently augment the type 2 immune response. In certain situations, they have been demonstrated to restrain the type 2 response by upregulating the expression of neuromedin B (NMB) receptors on ILC-2 cells during *N. brasiliensis* infection. NMB significantly reduce production of IL-5 and IL-13 by ILC2s [89].

### 4.4. Eosinophils

Eosinophils constitute 5% of leukocytes [90]. Similar to basophils, eosinophils exit the bone marrow fully differentiated, circulate in the bloodstream, and migrate to sites of inflammation in response to eotaxin-1/2/3 or IL-5 [91]. Eosinophils are consistently present throughout the gastrointestinal tract, except for the esophagus. They have a short half-life in the blood (8–18 h), but their lifespan in tissues ranges from 2 to 5 days. In vitro studies have shown that certain cytokines can prolong their lifespan for up to 14 days [92]. Eosinophil differentiation occurs in the bone marrow due to the exposure of progenitor cells to cytokines such as IL-5, IL-3, and GM-CSF. However, IL-5 plays a pivotal role in eosinophil differentiation and the expansion of eosinophil progenitor cells. It is also responsible for increasing their sensitivity to eotaxin-1 and enhancing eosinophil survival in the mucosa during type 2 inflammation [93].

Eosinophils display a variety of receptors on their surface, with the key players in their activation being the receptors for interleukin-5 (IL-5R) and CC-chemokine receptor 3 (CCR3), which mediate the chemotaxis of eosinophils in response to eotaxins. Additionally, eosinophils express immunoglobulin receptors, including the high-affinity immunoglobulin E receptor (FcεRI), low-affinity immunoglobulin G receptor (FcγRII), and immunoglobulin A receptor (FcαR). Interestingly, mouse eosinophils do not express FcεRI [94]. Furthermore, eosinophils express various families of PRRs, such as retinoic acid-inducible gene-like receptors (RIG-like), NLRs, and TLRs. However, the expression of TLRs in eosinophils is lower compared to neutrophils and macrophages. Among the Toll-like receptors expressed by eosinophils, TLR7 is the most prominent. Located in endosomes, TLR7 is responsible for detecting ssRNA [95]. Its activation plays a crucial role in regulating the adhesion, migration, and chemotactic responses of eosinophils, thereby contributing to the prolonged survival of these cells [96].

Activated eosinophils discharge cytotoxic granules containing proteins such as major basic protein (MBP-1 and MBP-2), eosinophil peroxidase (EPO), eosinophil-derived neurotoxin (EDN), and eosinophil cationic protein (ECP), alongside cytokines like IL-4, IL-6, IL-10, IL-13, TGF-β, and VEGF. The MBP proteins induce cytotoxicity in cells by disrupting the lipid bilayer and enhancing hydrogen peroxide production by macrophages, with MBP-1 exhibiting stronger activity than MBP-2. EDN and ECP are categorized as ribonucleases; however, EDN displays lower toxicity towards parasites and is more effective against single-stranded RNA viruses. EPO produces potent oxidizing substances, such as hypochlorous acid, exerting cytotoxic effects on parasites [97]. These granules are released through three mechanisms: classical exocytosis (where all granules are released without cell lysis), cytolysis with granule release, and partial degranulation (PMD). The predominant physiological mechanism for releasing eosinophil granule-stored proteins is PMD [98].

The role of eosinophils in protective responses against helminths seems to vary depending on both the host and the specific helminth involved [99]. Additionally, despite the association of parasitic infections with eosinophilia, there are helminth infection models in which a reduction in eosinophil numbers does not significantly impact the initiation of Th_2_ responses [100]. Consequently, the significance of eosinophils in protective responses against helminths may be overstated, and additional research is required to attain a more comprehensive understanding of this subject.

### 4.5. Neutrophils

Neutrophils are the most prevalent (50–70%) type of leukocytes in humans, and they have a relatively short half-life in the bloodstream, typically less than 6–8 h [101]. Due to their strong phagocytic capabilities, neutrophils are primarily associated with immune responses against bacteria and viruses. However, their role in defending against parasites that harbor bacteria may be underappreciated. Metazoan parasites, apart from causing evident physical tissue damage, can also act as carriers for potentially harmful bacteria that trigger a Th_1_ immune response, thus dampening the protective Th_2_ response to helminths. Neutrophils participate in neutralizing the bacteria carried by these parasites, indirectly contributing to helminth infection termination [102], especially since some bacteria are symbionts for parasites [103,104]

Furthermore, in vitro investigations have revealed that neutrophils possess the capacity to directly combat multicellular parasites [64]. They may release myeloperoxidase (MPO), which catalyzes the generation of reactive oxygen intermediates and plays a role in killing worm larvae. Studies have indicated that neutrophil-derived MPO is even more effective than eosinophil peroxidase in targeting *T. spiralis* larvae [105]. Another mechanism that neutrophils use to counteract multicellular parasites is the production of extracellular traps known as neutrophil extracellular traps (NETs). NETs are mesh-like structures composed of nuclear DNA, histones, and antimicrobial peptides that are released into the extracellular environment to ensnare and immobilize microorganisms such as bacteria, fungi, and parasites. The physical barrier created by NETs prevents the spread of pathogens and facilitates their subsequent elimination by other immune cells.

It is intriguing that neither macrophages nor neutrophils, when acting individually, are capable of eliminating the helminth [106]. Neutrophils could potentially play a role in fostering the Th_2_ response by generating Ym1, which has an inhibitory effect on IFN-γ. Conversely, Th_2_ cytokines appear to inhibit NET formation and neutrophil recruitment [107], which appears to conflict with their role in helminth defense. Nonetheless, Heeb et al. propose that this does not necessarily exclude the potential involvement of neutrophils in the defense against helminths, considering their high numbers in the bloodstream and rapid mobilization for pathogen combat [107].

During *N. brasiliensis* infection, a distinct early influx of neutrophils into the lungs is observed just two days after infection [108]. Likewise, during the Th_2_ response induced by *H. polygyrus* infection, neutrophils gather around the worms and penetrate their mucosal and submucosal layers [109]. This implies they are among the first immune cells to react to parasite infections, acting as the primary line of defense before the more targeted Th_2_ response develops.

Additionally, in vitro experiments suggest that, like macrophages and lymphocytes, neutrophils can alter their phenotype in response to the cytokine milieu. During helminth infections, neutrophils may exhibit the expression of IL-13 and IL-33 and fall into the category of N2 neutrophils, although further in vivo experiments are necessary to validate this occurrence [110]. In summary, while the precise role of neutrophils in the response against helminths is not yet fully understood, it can tentatively be concluded that they play a supporting role in the initial phases of infection, targeting the bacteria harbored by helminths. They also potentially play an auxiliary role in later stages through NET formation.

### 4.6. Group 2 Innate Lymphoid Cells (ILC2s)

Group 2 innate lymphoid cells (ILC2s) were discovered in adipose tissue in 2010 [111], and subsequent research has unveiled their abundance in mucosal membranes throughout different organs. In vitro studies have indicated that ILC2s derived from adipose tissue can survive up to 18 months in the presence of IL-2 [112]. These cells demonstrate a swift and potent reaction to a diverse array of environmental factors, including cytokines, neurotransmitters, hormones, nutrients, and lipid mediators, effectively coordinating Th_2_ responses.

**Figure 3:**
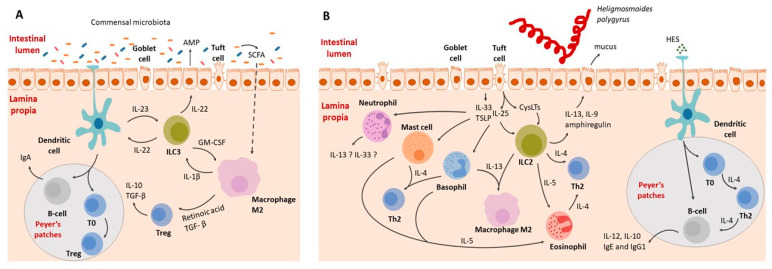
Schematic representation of the interaction between the intestinal immune system during homeostasis (**A**) and helminth-induced inflammation (**B**). (**A**) Interactions between the immune system and gut microbiota. Dendritic cells function through both antigen presentation to naive B and T cells and by secreting IL-23, activating type 3 innate lymphoid cells (ILC3). Activated ILC3s release IL-22 and granulocyte–macrophage colony-stimulating factor (GM-CSF). IL-22 activates the epithelium, leading to the secretion of antimicrobial peptides (AMP) and an increase in IL-23 production by dendritic cells. Bacterial metabolites also directly interact with the immune system. For example, short-chain fatty acids (SCFAs) penetrate the epithelial barrier and upregulate anti-inflammatory cytokines. T regulatory cells are crucial for maintaining tolerance towards commensal microbiota. (**B**) Immune system–helminth interactions, illustrated using the example of *H. polygyrus*. Intestinal parasites release enzymes that digest the mucosal barrier, causing epithelial cell death. In response to the damage, intestinal epithelial cells release alarmins (IL-25, IL-33, TSLP), activating immune system cells. Alarmins activate basophils, mast cells, ILC2s, and neutrophils. Subsequently, eosinophils are activated by IL-5, Th2 cells by IL-4, and type 2 macrophages by IL-13. The role of neutrophils in the development of type 2 responses is not fully understood yet, but there is a suspicion that they may be capable of secreting IL-13 and IL-33. Other cells contribute to the inflammatory response, recruitment of immune cells, and coordination of the immune reaction.

ILC2s activation is predominantly initiated by IL-33, IL-25, or TSLP, which are secreted by damaged epithelial cells (Figure 3). However, they are also responsive to a range of other factors, including IL-4, IL-9, prostaglandin D2 (PGD2), cysteinyl leukotrienes (CysLT), neuropeptides such as neuromedin U, vasoactive intestinal peptide (VIP), acetylcholine, calcitonin gene-related peptide (CGRP), androgens, and estrogens [113].

Upon activation, ILC2s release a variety of cytokines, including IL-5, IL-13, IL-9, amphiregulin, and small amounts of IL-4. Production of these cytokines leads to alterations in the gut epithelium and the activation of various immune cells, including dendritic cells (DCs), eosinophils, B cells, macrophages, Th_2_ cells, and mast cells [114]. ILC2s, being the main source of IL-13, play a crucial role in regulating the Th_2_ response. The significance of IL-13 has been demonstrated in mice infected with *Nippostrongylus brasiliensis*, where animals deprived of IL-13 showed compromised parasite expulsion [115].

Additionally, the capacity of ILC2s to coordinate immune responses might be even more significant, as ILC2s found in the spleen and lymph nodes express MHCII and can present antigens, allowing for the expansion of CD4^+^ T cells [116].

### 4.7. Mast Cells (MC)

The term “mast cell” is derived from the German word “mastung”, which translates to “well-nourished”. This term was coined in the late 19th century by the German scientist Paul Ehrlich, who observed plentiful granules in the cytoplasm of these cells [117]. Mast cells, often abbreviated as MCs, are myeloid immune cells situated in the connective tissue of mucosal membranes, particularly in the subepithelial regions and around blood vessels. In contrast to other hematopoietic cells, they complete their maturation in peripheral tissues rather than in the bone marrow. Mast cells have a relatively extended lifespan, with the potential to persist for up to 12 weeks [118]. They tend to accumulate in inflamed tissues and can be activated by various cytokines, including stem cell factor (SCF), IL-3, IL-4, IL-9, and IL-33, as well as IgE class antibodies [119].

Stimulation of the FcεRI receptor on mast cells initiates degranulation, resulting in the release of a diverse range of preformed bioactive substances, including histamine, serotonin, IL-4, IL-5, VEGF, TNF, and proteases [120], along with the de novo production of immune mediators like IL-33, prostaglandin D2, and leukotriene C4 [121]. Despite extensive research endeavors, the intricate nature of the substances released and the processes involved continues to present challenges in elucidating the exact role of mast cells in the immune response.

Mast cells experience degranulation in the early stages of parasitic worm infections, and this occurs regardless of IgE, facilitating the attraction and expansion of neutrophils, the induction of M2 [122], and the modulation of dendritic cell activity through histamine release [123].

### 4.8. T Lymphocytes

T helper lymphocytes, often referred to as CD4^+^ T cells, are crucial in coordinating the adaptive immune response. Upon leaving the thymus, T cells are referred as Th_0_ cells. Then, they migrate to the spleen and lymph nodes, where they are activated by antigens presented by DC (dendritic cells) complexed with MHCII. This is followed by differentiation into distinct subtypes (Th_1_, Th_2_, Th_9_, Th_17_, Th_22_, and T_reg_ cells) depending on the antigen properties and cytokine milieu [124]. Each cell population is responsible for a different action of the immune system. In general, Th1/Th_17_ is associated with cell-mediated immunity and inflammation, targeting bacteria and viruses. The Th_2_ response is supposed to combat multicellular invaders and is associated with IL-4, IL-5, IL-9, and IL-13, with hallmarks of eosinophils, basophils, M2 activation, tissue regeneration (Figure 3), and an antibody class switching from IgM to IgE and IgG1 in mice [125].

Although the precise factors responsible for differentiations into particular phenotypes are not fully defined, it is well established that Th_2_ cells, which are crucial for expelling helminths, differentiate in the presence of IL-4 [126]. This process is tightly regulated by transcription factors such as GATA3 [127], STAT5 [128], and STAT6 [129]. The role of transcription factors can vary in terms of inducing and maintaining differentiation. For instance, mice lacking Gata3 are incapable of generating Th_2_ cells. Removing Gata3 from fully differentiated Th_2_ cells has only a minimal impact on IL-4 production but completely blocks IL-5 and IL-13 production [130]. While low STAT5 expression is sufficient for cell proliferation and survival, robust STAT5 signaling is necessary for Th_2_ differentiation and the subsequent release of the characteristic Th_2_ cytokine profile, which includes IL-4, IL-5, IL-9, and IL-13 [131].

The development of the Th_2_ response is also supported by T follicular helper (T_fh_) cells, which play a key role in presenting antigens to B cells within germinal centers. They provide essential signals to activated B cells, promoting their development, selection, and antibody release [132]. T_fh_ cells may also release IL-4, but unlike Th_2_ cells, they do not require IL-4 for their differentiation or to initiate and maintain the immune response against helminths. However, to ensure a balanced and long-lasting response, which, when excessive, can be detrimental, regulation is necessary. This regulation is achieved through the development of another T cell population, T_reg_ cells, which differentiate under the control of Foxp3 [133] and STAT5 transcription factors [134].

T_reg_ cells can be categorized into two main types: natural T_reg_ (nT_reg_) cells, which originate in the thymus, and induced T_reg_ (iT_reg_) cells, which differentiate in peripheral tissues from naive T cells under the influence of IL-2 or TGF-β. Both of these T_reg_ cell populations serve to suppress immune responses, which is beneficial for helminths, as it allows them to persist within the host’s body [135].

One of the critical roles of T_reg_ cells is to shift the production of antibody classes by B cells from IgE to IgG4 [136] in humans (or the equivalent in mice lacking IgG4). IgG4 and IgE compete for epitopes, and elevated IgG4 levels prevent immune IgE from activating eosinophils and basophils, thus regulating immune responses [137]. IgE plays a role in the immune response to helminths by triggering the release of histamine and other mediators, promoting Th2 inflammation, and facilitating parasite expulsion. In contrast, IgG4 suppresses parasite-specific T cell proliferation, reduces levels of Th2 cytokines, and abolishes Th1 cytokines [138].

Given T_reg_ cells’ ability to dampen immune responses, they offer distinct advantages to helminths. In mice infected with *H. polygyrus*, T_reg_ cell proliferation is particularly pronounced in close proximity to the parasite, notably within Peyer’s patches (PP). This strongly suggests that substances released by the nematode may act locally to stimulate T_reg_ cell expansion. *H. polygyrus* releases a TGF-β mimic (*Hp*-TGM), one of the proteins likely responsible for this T_reg_ cell amplification, which effectively mimics the function of TGF-β, a pivotal factor in T_reg_ cell differentiation. It also serves as a potent inducer of both mouse and human Foxp3+ T_reg_ cells in vitro [139].

During the progression of a helminth infection, T_reg_ cells can be induced through several mechanisms:

- Early expansion of natural T_reg_ cells occurring 3–7 days after infection, followed by the induction of T_reg_ cells.
- Directly by ES products of the helminth.
- Indirectly through the stimulation of dendritic cells (DCs), which in turn induce T_reg_ cell expansion.
- Through the action of M2 macrophages, which differentiate in response to IL-33 released upon epithelial damage [140].

### 4.9. B Lymphocytes

B cells represent a subgroup of lymphocytes responsible for generating antibodies that target invading pathogens. During Th_2_ immune responses, B cells release antibodies belonging to the IgE and IgG1 classes. IgE antibodies effectively coat parasites, essentially marking them as targets for basophils and mast cells, which subsequently trigger degranulation. However, the effectiveness of the antibody response against helminths varies significantly and is species-dependent. For example, IgE-dependent responses do not play a significant role in infections with *H. polygyrus* [141] or *S. mansoni* [142] in mice. Nonetheless, during reinfection with *H. polygyrus*, B lymphocytes become crucial for expelling the parasite, and IgG is associated with immunity [143]. Although the exact mechanisms behind this phenomenon are not fully understood, the antibody-dependent immunity upon secondary exposure may be linked to increased antibody production and higher affinity. This leads not only to opsonization but also to interference with parasite migration, potentially by disrupting chemosensory reception [144].

Furthermore, antibodies play a significant role in granulomas, which are cell aggregates that surround the parasite. Although lymphocytes are rare in granulomas, antibodies are crucial in these structures [145]. IgG binds to FcGR receptors on basophils, eosinophils, mast cells, and macrophages, resulting in antibody-dependent cytotoxicity, the release of cytokines and chemokines, and cell degranulation [146].

B cells can also play a regulatory role in immune responses by inhibiting dendritic cells’ (DCs) ability to produce IL-12, which is associated with the Th_1_ response. This indirect action promotes the Th_2_ response [147]. Additionally, there exists a subpopulation of B_reg_ lymphocytes that produce IL-10, which inhibits Th_2_ differentiation. Furthermore, the absence of B lymphocytes during schistosomiasis in mice disrupts regulatory mechanisms, leading to an excessive Th_2_ polarization [148].

## 5. Granulomas

Certain helminths, such as *T. canis*, *S. japonicum*, and *H. polygyrus*, have the ability to trigger an immune response that results in the formation of a specific structure known as a granuloma. A granuloma is a well-organized, compact structure that develops during extended immune responses to persistent pathological stimuli that cannot be eradicated through acute inflammatory processes. The term “granuloma” was first coined in the latter half of the 19th century to describe changes in lungs affected by tuberculosis [149]. Over time, it became evident that granulomas form not only in response to bacterial infections like tuberculosis but also in the presence of other pathogens (viruses, parasites, and fungi), as well as non-infectious agents, such as autoimmune diseases like Crohn’s disease and foreign bodies [150]. One distinctive feature of granulomas, in contrast to typical chronic inflammatory infiltrates, is the characteristic organization of specialized forms of macrophages: epithelioid cells and giant cells.

### 5.1. Structure of a Granuloma

During parasitic infections, granulomas can include all the cell types associated with type 2 inflammation, in addition to specialized macrophages like epithelioid cells and giant cells, along with fibroblasts. The term “epithelioid cells” is derived from their resemblance to epithelial cells. These cells closely adhere to one another, providing a barrier function, and they have a distinctive flattened appearance with elongated nuclei. Giant cells, which have three or more cell nuclei, play a phagocytic role within granulomas. An important component of granulomas is also fibroblasts, which are spindle-shaped cells responsible for synthesizing extracellular matrix components like collagen and elastic fibers [151]. The specific cellular composition of granulomas can vary depending on the causative factor, location, and host.

The structure of granulomas not only depends on the class of pathogen (virus, bacteria, helminth) but also on the particular species of the pathogen. In the case of *H. polygyrus* infection, granulomas directly envelop the parasite. They primarily consist of neutrophils, encircled and dominated by macrophages (including epithelioid and giant cells), while Th lymphocytes, dendritic cells, and eosinophils are observed at the periphery of these structures. These granulomas do not contain basophils, mast cells, or B lymphocytes [152]. In the case of another nematode, *Toxocara canis*, in the first 10 days post-infection, the granuloma is primarily composed of eosinophils (60–80%), with the remaining cells being macrophages (giant cells), lymphocytes, and a small number of neutrophils [153] By day 28 post-infection, the granuloma takes on a different form. It contains fewer cells, with macrophages and epithelial cells in the central area, and peripherally, there are fibroblasts and scattered eosinophils [154]. For *S. japonicum* infection, neutrophils are the most abundant cell type, while *S. mansoni* granulomas are characterized by extensive eosinophil infiltrates [155].

The location within the host’s body where granulomas form also influences the cellular composition of these structures. Histological examination of mice infected with *S. mansoni* has revealed distinctions between colonic and hepatic granulomas. Colonic granulomas contain more macrophages but fewer collagen fibers, eosinophils, and T and B lymphocytes; nevertheless, the cells are more organized. These differences may be linked to the distinct fates of the eggs in these organs. In the intestines, eggs are expelled with feces, which is not the case for eggs located in the liver [156].

Furthermore, it has been noted that the composition of granulomas can differ among various strains of laboratory mice. Specifically, both the CBA and C3H mouse strains tend to exhibit a more severe progression of schistosomiasis compared to the BALB/c and C57BL/6 strains. In CBA and C3H mice, there is a greater degree of splenomegaly, and the liver granulomas are less well-defined. The underlying reasons for the substantial variations in immunopathology among different mouse strains remain undefined, mainly due to the fact that studies are generally conducted using a single strain [157].

### 5.2. Granuloma Formation during Parasitic Infections

**Figure 4:**
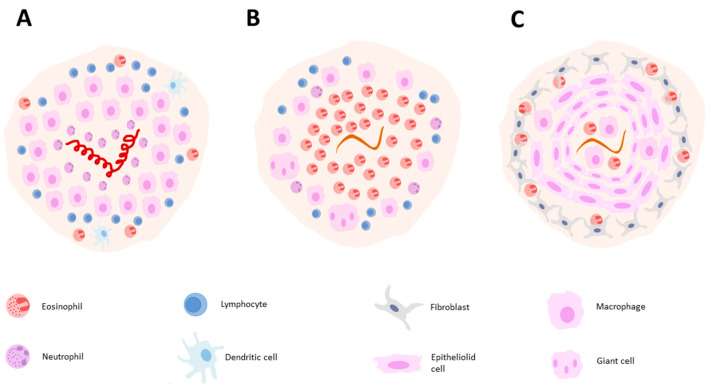
Illustration of granuloma composition in *H. polygyrus* 4 days post-infection (**A**). *T. canis* 10 days post-infection (**B**) and *T. canis* 28 days post-infection (**C**).

Granulomas exhibit a complex structure (Figure 4), determined by the parasitic species and the tissue in which they form. A significant element of granulomas comprises epithelioid and giant cells, which originate from macrophages. The formation of epithelioid cells is well-documented and is triggered by IL-4 or IL-13, leading to STAT6-dependent transcription of genes encoding E-cadherin, a cell–cell adhesion protein [158]. The formation of giant cells has been a subject of debate for some time. Previously, it was suggested that they are formed through the fusion of macrophages, similar to the pathway for epithelioid cell formation [159,160]. However, recent studies indicate that M-CSF, in combination with parasite-derived factors, induces the proliferation of myeloid bone marrow precursors, followed by their differentiation into polyploid giant cells [161]. The increased chromosome number is linked to the failure of cytokinesis during cell division, resulting from the induction of replicative stress. Nevertheless, the second hypothesis does not exclude the first one, as monocytes can transform into giant cells through cell fusion when cultured with mycobacterial glycoproteins without the addition of CSF [162]. Over time, granulomas undergo fibrosis, characterized by the excessive deposition of connective tissue. This is associated with the Th_2_ cytokine-induced environment created by the parasite, leading to the activation of fibroblasts and the production of extracellular matrix components.

The cytokine environment exerts a profound influence on the granuloma structure. During *H. polygyrus* infection, granuloma formation is primarily orchestrated by the induction of a Th_2_ immune response [163]. In contrast, the process of granuloma formation during *S. mansoni* infection is more intricate, involving multiple stages of the immune response, likely since the fluke navigates through tissues, engaging various immune mechanisms. Schistosomes deposit eggs in the blood vessels close to the endothelium, where endothelial cells envelop the egg to facilitate its expulsion. Following the egg’s exit from the blood vessels, a granuloma forms around it [156]. When assessing granuloma development in the liver during *S. mansoni* infection, we can discern four distinct phases. The initial phase, referred to as the pre-granulomatous exudative phase, entails an unorganized colonization by the initial inflammatory cells around the parasite’s egg. The necrotic–exudative phase displays a more complex array of inflammatory cells, distributed unevenly across different layers, with central necrosis. The exudative–productive phase showcases a rich structure of collagen fibers, with inflammatory cells concentrated at the periphery. Lastly, the productive phase features prominent thick bands of collagen fibers between the egg and a limited number of inflammatory cells [164].

The immune response evolves as the infection progresses, thereby affecting the composition of granulomas. Initially, a Th_1_ response dominates, characterized by elevated levels of interleukin-2 (IL-2) and interferon-gamma (IFN-γ), persisting until the egg-laying phase (approximately 5 weeks after infection). In the subsequent 1–2 weeks, granuloma formation intensifies due to a shift toward a Th_2_ response, marked by the presence of IL-4 and IL-5. During the chronic phase, which extends for over 3 months, the intensity of the Th_2_ response diminishes and regulatory T and B lymphocytes emerge, resulting in a state of reduced immune system reactivity [157]. During schistosomiasis, IL-13 takes on a prominent role in this process, while IL-4 plays a supportive role [165]. IL-13 plays a significant role in promoting collagen synthesis in fibroblasts by inducing the expression of arginase. Arginase is involved in the conversion of L-arginine into proline, a crucial building block for collagen [166].

### 5.3. Function of Granulomas

Granulomas have a crucial impact on the overall health of the host. They serve to protect healthy host tissues from the harmful effects of eggs and parasites and prevent interactions with toxic excretory–secretory products like the hepatotoxic omega-1 and IPSE/alpha-1 released by Schistosome eggs. However, the process of granuloma formation is associated with stress and chronic inflammation.

Certain substances, such as β-glucuronidase, leukotrienes, and free radicals produced by cell populations within the granuloma, have the potential to cause damage to the surrounding tissue. Therefore, the formation of granulomas is under the regulation of T and B lymphocytes to limit excessive tissue damage [167].

Furthermore, the development of fibrosis around the eggs can be detrimental to the host and may culminate in the progression of chronic hepatic schistosomiasis. Approximately 5 to 15 years following infection with *S. mansoni*, collagen deposits can accumulate in periportal spaces, causing blockage of blood flow, portal hypertension, and the emergence of esophageal varices [168]. The rupture of these varices can result in bleeding from the esophagus [169]. This highlights the fact that granulomas represent a sort of compromise—they enable the host to survive with the infection for many years, but simultaneously, they are associated with chronic and adverse effects [170].

Indeed, *Schistosoma mansoni* effectively manipulates the host’s immune response to aid in the expulsion of its eggs, a crucial step for the completion of its life cycle. This has been substantiated by research, such as the study conducted by Dunne et al. in 1983, which demonstrated that mice subjected to thymectomies expelled fewer eggs compared to control mice with an intact immune system [164]. The thymectomized mice had lower egg counts both in their intestines and feces [171]. Numerous researchers have also proposed that granulomas play a beneficial role for the parasite, and the inflammatory response within these granulomas in the intestines facilitates the translocation of eggs into the lumen and their subsequent excretion [172,173,174]. The eggs of the parasite, found in the feces of infected humans as well as experimental mice, lack an outer granulomatous layer, suggesting that the eggs exit the granuloma before reaching the intestinal lumen. However, the precise mechanism by which granulomas facilitate the passage of eggs into the intestinal lumen and how the eggs exit the granuloma remains to be fully determined.

A recent study conducted by Takaki et al. (2021) offers intriguing insights into this process. The study revealed that immature *S. mansoni* eggs possess an immunologically neutral shell that enables them to remain concealed from the host’s immune system [175]. The actual immune response is initiated when the miracidium inside the egg reaches maturity, subsequently recruiting macrophages and leading to the formation of granulomas. The study also noted the presence of both immature and mature eggs in the intestinal wall, with only mature eggs observed in the mouse feces [175]. These findings raise questions regarding the factors that determine the immunological neutrality of eggs and the precise role of granulomas in this process. Further research is essential to gain a comprehensive understanding of the intricate interplay between the parasite, the host immune system, and the function of granulomas in facilitating egg excretion.

## 6. Conclusions

A number of cell populations (both hematopoietic and non-hematopoietic-derived) participate in recognizing helminth infection and raising and sustaining the immune response. The interactions between these cells are complicated and not fully understood, especially those depending on the host and the invader species. However, their role may be pivotal. An efficient response against a helminth species may depend on particular cells, whereas this population may be of little significance in combating other helminth species. There is significant work to be done in deciphering these interactions and their underlying mechanisms; nevertheless, the existing knowledge allows us to provide a foundation for the development of methods that will allow us to both combat these infections and use them to mitigate autoimmune diseases and allergies in the future.

## Data Availability

Not applicable.

## References

[1] Hotez P.J., Brindley P.J., Bethony J.M., King C.H., Pearce E.J., Jacobson J. (2008). Helminth infections: The great neglected tropical diseases. J. Clin. Investig..

[2] https://www.cdc.gov/parasites/about.html.

[3] Cox F.E. (2002). History of human parasitology. Clin. Microbiol. Rev..

[4] Gross M.D. (2010). Chasing Snails: Anti-Schistosomiasis Campaigns in the People’s Republic of China.

[5] Berry-Cabán C.S. (2007). Return of the God of plague: Schistosomiasis in China. J. Rural Trop. Public Health.

[6] https://www.cdc.gov/parasites/sth/index.html.

[7] https://www.who.int/health-topics/lymphatic-filariasis#tab=tab_1.

[8] https://www.who.int/health-topics/schistosomiasis#tab=tab_1.

[9] https://apps.who.int/neglected_diseases/ntddata/sth/sth.html..

[10] Pullan R.L., Smith J.L., Jasrasaria R., Brooker S.J. (2014). Global numbers of infection and disease burden of soil transmitted helminth infections in 2010. Parasites Vectors.

[11] Kim C.L., Agampodi S., Marks F., Kim J.H., Excler J.L. (2023). Mitigating the effects of climate change on human health with vaccines and vaccinations. Front. Public Health.

[12] Alsarraf M., Carretón E., Ciuca L., Diakou A., Dwużnik-Szarek D., Fuehrer H.P., Genchi M., Ionică A.M., Kloch A., Kramer L.H. (2023). Diversity and geographic distribution of haplotypes of Dirofilaria immitis across European endemic countries. Parasites Vectors.

[13] Jia T.W., Melville S., Utzinger J., King C.H., Zhou X.N. (2012). Soil-transmitted helminth reinfection after drug treatment: A systematic review and meta-analysis. PLoS Neglected Trop. Dis..

[14] Wiśniewski M., Jaros S., Bąska P., Cappello M., Wędrychowicz H. (2013). Ancylostoma ceylanicum metalloprotease 6 DNA vaccination induces partial protection against hookworm challenge infection. Acta Parasitol..

[15] Wiśniewski M., Jaros S., Bąska P., Cappello M., Długosz E., Wędrychowicz H. (2016). Hamsters vaccinated with Ace-mep-7 DNA vaccine produced protective immunity against Ancylostoma ceylanicum infection. Exp. Parasitol..

[16] Buffoni L., Piva M.M., Baska P., Januszkiewicz K., Norbury L.J., Prior K.C., Dezen D., Silva A.S., Wedrychowicz H., Mendes R.E. (2020). Immunization with the recombinant myosin regulatory light chain (FhrMRLC) in Adjuplex^®^ adjuvant elicits a Th1-biased immune response and a reduction of parasite burden in Fasciola hepatica infected rats. Parasitol. Int..

[17] Wesołowska A., Basałaj K., Norbury L.J., Sielicka A., Wędrychowicz H., Zawistowska-Deniziak A. (2018). Vaccination against Fasciola hepatica using cathepsin L3 and B3 proteases delivered alone or in combination. Vet. Parasitol..

[18] Wilson R.A. (2023). Models of Protective Immunity against Schistosomes: Implications for Vaccine Development. Pathogens.

[19] Wong M.T., Anuar N.S., Noordin R., Tye G.J. (2023). Soil-transmitted helminthic vaccines: Where are we now?. Acta Trop..

[20] Diemert D.J., Zumer M., Campbell D., Grahek S., Li G., Peng J., Bottazzi M.E., Hotez P., Bethony J. (2022). Safety and immunogenicity of the Na-APR-1 hookworm vaccine in infection-naïve adults. Vaccine.

[21] Queiroz-Glauss C.P., Vieira M.S., Gonçalves-Pereira M.H., Almeida S.S., Freire R.H., Gomes M.A., Alvarez-Leite J.I., Santiago H.C. (2022). Helminth infection modulates number and function of adipose tissue Tregs in high fat diet-induced obesity. PLoS Neglected Trop. Dis..

[22] Reyes J.L., Lopes F., Leung G., Mancini N.L., Matisz C.E., Wang A., Thomson E.A., Graves N., Gilleard J., McKay D.M. (2016). Treatment with cestode parasite antigens results in recruitment of CCR2+ myeloid cells, the adoptive transfer of which ameliorates colitis. Infect. Immun..

[23] Capron M., Béghin L., Leclercq C., Labreuche J., Dendooven A., Standaert A., Delbeke M., Porcherie A., Nachury M., Boruchowicz A. (2019). Safety of P28GST, a protein derived from a schistosome helminth parasite, in patients with Crohn’s disease: A pilot study (ACROHNEM). J. Clin. Med..

[24] Smits H.H., Hammad H., van Nimwegen M., Soullie T., Willart M.A., Lievers E., Kadouch J., Kool M., Oosterhoud J.K.-V., Deelder A.M. (2007). Protective effect of Schistosoma mansoni infection on allergic airway inflammation depends on the intensity and chronicity of infection. J. Allergy Clin. Immunol..

[25] Duan T., Du Y., Xing C., Wang H.Y., Wang R.-F. (2022). Toll-like receptor signaling and its role in cell-mediated immunity. Front. Immunol..

[26] Bermejo-Jambrina M., Eder J., Helgers L.C., Hertoghs N., Nijmeijer B.M., Stunnenberg M., Geijtenbeek T.B.H. (2018). C-type lectin receptors in antiviral immunity and viral escape. Front. Immunol..

[27] Babamale A.O., Chen S.-T. (2021). Nod-like receptors: Critical intracellular sensors for host protection and cell death in microbial and parasitic infections. Int. J. Mol. Sci..

[28] Kawasaki T., Kawai T. (2014). Toll-like receptor signaling pathways. Front. Immunol..

[29] Sameer A.S., Nissar S. (2021). Toll-like receptors (TLRs): Structure, functions, signaling, and role of their polymorphisms in colorectal cancer susceptibility. BioMed Res. Int..

[30] Babu S., Blauvelt C.P., Kumaraswami V., Nutman T.B. (2005). Diminished expression and function of TLR in lymphatic filariasis: A novel mechanism of immune dysregulation. J. Immunol..

[31] Babu S., Anuradha R., Kumar N.P., George P.J., Kumaraswami V., Nutman T.B. (2011). Filarial lymphatic pathology reflects augmented toll-like receptor-mediated, mitogen-activated protein kinase-mediated proinflammatory cytokine production. Infect. Immun..

[32] Pineda M.A., Eason R.J., Harnett M.M., Harnett W. (2015). From the worm to the pill, the parasitic worm product ES-62 raises new horizons in the treatment of rheumatoid arthritis. Lupus.

[33] Chen D., Zhao Y., Feng Y., Jin C., Yang Q., Qiu H., Xie H., Xie S., Zhou Y., Huang J. (2019). Expression of TLR2, TLR3, TLR4, and TLR7 on pulmonary lymphocytes of Schistosoma japonicum-infected C57BL/6 mice. Innate Immun..

[34] Thomas P.G., Carter M.R., Atochina O., Da’dara A.A., Piskorska D., McGuire E., Harn D.A. (2003). Maturation of dendritic cell 2 phenotype by a helminth glycan uses a Toll-like receptor 4-dependent mechanism. J. Immunol..

[35] Van Liempt E., van Vliet S.J., Engering A., Vallejo J.J., Bank C.M., Sanchez-Hernandez M., van Kooyk Y., van Die I. (2007). Schistosoma mansoni soluble egg antigens are internalized by human dendritic cells through multiple C-type lectins and suppress TLR-induced dendritic cell activation. Mol. Immunol..

[36] Paveley R.A., Aynsley S.A., Turner J.D., Bourke C.D., Jenkins S.J., Cook P.C., Martinez-Pomares L., Mountford A.P. (2011). The Mannose Receptor (CD206) is an important pattern recognition receptor (PRR) in the detection of the infective stage of the helminth Schistosoma mansoni and modulates IFNγ production. Int. J. Parasitol..

[37] Rodríguez E., Kalay H., Noya V., Brossard N., Giacomini C., van Kooyk Y., García-Vallejo J.J., Freire T. (2017). Fasciola hepatica glycoconjugates immuneregulate dendritic cells through the Dendritic Cell-Specific Intercellular adhesion molecule-3-Grabbing Non-integrin inducing T cell anergy. Sci. Rep..

[38] Favoretto B.C., Casabuono A.A., Portes-Junior J.A., Jacysyn J.F., Couto A.S., Faquim-Mauro E.L. (2017). High molecular weight components containing N-linked oligosaccharides of Ascaris suum extract inhibit the dendritic cells activation through DC-SIGN and MR. Mol. Immunol..

[39] Gringhuis S.I., Kaptein T.M., Wevers B.A., Mesman A.W., Geijtenbeek T.B.H. (2014). Fucose-specific DC-SIGN signalling directs T helper cell type-2 responses via IKKε-and CYLD-dependent Bcl3 activation. Nat. Commun..

[40] Geijtenbeek T.B.H., Gringhuis S.I. (2016). C-type lectin receptors in the control of T helper cell differentiation. Nat. Rev. Immunol..

[41] Pellefigues C., Tang S.-C., Schmidt A., White R.F., Lamiable O., Connor L.M., Ruedl C., Dobrucki J., Le Gros G., Ronchese F. (2017). Toll-like receptor 4, but not neutrophil extracellular traps, promote IFN type I expression to enhance Th2 responses to Nippostrongylus brasiliensis. Front. Immunol..

[42] Thawer S., Auret J., Schnoeller C., Chetty A., Smith K., Darby M., Roberts L., Mackay R.M., Whitwell H.J., Timms J.F. (2016). Surfactant protein-D is essential for immunity to helminth infection. PLoS Pathog..

[43] Reynolds L.A., Harcus Y., Smith K.A., Webb L.M., Hewitson J.P., Ross E.A., Brown S., Uematsu S., Akira S., Gray D. (2014). MyD88 signaling inhibits protective immunity to the gastrointestinal helminth parasite Heligmosomoides polygyrus. J. Immunol..

[44] Hang L., Blum A.M., Kumar S., Urban J.F., Mitreva M., Geary T.G., Jardim A., Stevenson M.M., Lowell C.A., Weinstock J.V. (2016). Downregulation of the Syk signaling pathway in intestinal dendritic cells is sufficient to induce dendritic cells that inhibit colitis. J. Immunol..

[45] Alhallaf R., Agha Z., Miller C.M., Robertson A.A., Sotillo J., Croese J., Cooper M.A., Masters S.L., Kupz A., Smith N.C. (2018). The NLRP3 inflammasome suppresses protective immunity to gastrointestinal helminth infection. Cell Rep..

[46] Celias D.P., Motrán C.C., Cervi L. (2020). Helminths turning on the NLRP3 inflammasome: Pros and cons. Trends Parasitol..

[47] Luo X.C., Chen Z.H., Xue J.B., Zhao D.X., Lu C., Li Y.H., Li S.M., Du Y.W., Liu Q., Wang P. (2019). Infection by the parasitic helminth Trichinella spiralis activates a Tas2r-mediated signaling pathway in intestinal tuft cells. Proc. Natl. Acad. Sci. USA.

[48] Mabbott N.A., Donaldson D.S., Ohno H., Williams I.R., Mahajan A. (2013). Microfold (M) cells: Important immunosurveillance posts in the intestinal epithelium. Mucosal Immunol..

[49] Sánchez-Quintero A., Bradford B.M., Maizels R., Donaldson D.S., Mabbott N.A. (2019). Effect of co-infection with a small intestine-restricted helminth pathogen on oral prion disease pathogenesis in mice. Sci. Rep..

[50] Selleri S., Palazzo M., Deola S., Wang E., Balsari A., Marincola F.M., Rumio C. (2008). Induction of pro-inflammatory programs in enteroendocrine cells by the Toll-like receptor agonists flagellin and bacterial LPS. Int. Immunol..

[51] Palazzo M., Balsari A., Rossini A., Selleri S., Calcaterra C., Gariboldi S., Zanobbio L., Arnaboldi F., Shirai Y.F., Serrao G. (2007). Activation of enteroendocrine cells via TLRs induces hormone, chemokine, and defensin secretion. J. Immunol..

[52] Daly K., Burdyga G., Al-Rammahi M., Moran A., Eastwood C., Shirazi-Beechey S. (2020). Toll-like receptor 9 expressed in proximal intestinal enteroendocrine cells detects bacteria resulting in secretion of cholecystokinin. Biochem. Biophys. Res. Commun..

[53] Yu Y., Yang W., Li Y., Cong Y. (2020). Enteroendocrine cells: Sensing gut microbiota and regulating inflammatory bowel diseases. Inflamm. Bowel Dis..

[54] Ovington K.S., Bacarese-Hamilton A.J., Bloom S.R. (1985). Nippostrongylus brasiliensis: Changes in plasma levels of gastrointestinal hormones in the infected rat. Exp. Parasitol..

[55] Worthington J.J., Samuelson L.C., Grencis R.K., McLaughlin J.T. (2013). Adaptive immunity alters distinct host feeding pathways during nematode induced inflammation, a novel mechanism in parasite expulsion. PLoS Pathog..

[56] Thomas P.A., Akwari O.E., Kelly K.A. (1979). Hormonal control of gastrointestinal motility. World J. Surg..

[57] Peikin S.R. (1989). Role of cholecystokinin in the control of food intake. Gastroenterol. Clin. N. Am..

[58] Bąska P., Zawistowska-Deniziak A., Norbury L.J., Wiśniewski M., Januszkiewicz K. (2019). Fasciola hepatica isolates induce different immune responses in unmaturated bovine macrophages. J. Vet. Res..

[59] Bąska P., Norbury L.J., Zawistowska-Deniziak A., Wiśniewski M., Januszkiewicz K. (2017). Excretory/secretory products from two Fasciola hepatica isolates induce different transcriptional changes and IL-10 release in LPS-activated bovine “BOMA” macrophages. Parasitol. Res..

[60] Gerbe F., Sidot E., Smyth D.J., Ohmoto M., Matsumoto I., Dardalhon V., Cesses P., Garnier L., Pouzolles M., Brulin B. (2016). Intestinal epithelial tuft cells initiate type 2 mucosal immunity to helminth parasites. Nature.

[61] Rajeev S., Sosnowski O., Li S., Allain T., Buret A.G., McKay D.M. (2021). Enteric tuft cells in host-parasite interactions. Pathogens.

[62] Strine M.S., Wilen C.B. (2022). Tuft cells are key mediators of interkingdom interactions at mucosal barrier surfaces. PLoS Pathog..

[63] Haber A.L., Biton M., Rogel N., Herbst R.H., Shekhar K., Smillie C., Burgin G., Delorey T.M., Howitt M.R., Katz Y. (2017). A single-cell survey of the small intestinal epithelium. Nature.

[64] Westphalen C.B., Asfaha S., Hayakawa Y., Takemoto Y., Lukin D.J., Nuber A.H., Brandtner A., Setlik W., Remotti H., Muley A. (2014). Long-lived intestinal tuft cells serve as colon cancer–initiating cells. J. Clin. Investig..

[65] Bąska P., Norbury L.J. (2022). The Role of the Intestinal Epithelium in the “Weep and Sweep” Response during Gastro—Intestinal Helminth Infections. Animals.

[66] McGinty J.W., Ting H.A., Billipp T.E., Nadjsombati M.S., Khan D.M., Barrett N.A., Liang H.E., Matsumoto I., von Moltke J. (2020). Tuft-cell-derived leukotrienes drive rapid anti-helminth immunity in the small intestine but are dispensable for anti-protist immunity. Immunity.

[67] Jiang W., Wang Z., Zhang J., Li M. (2022). Interleukin 25 and its biological features and function in intestinal diseases. Cent. Eur. J. Immunol..

[68] Su J., Chen T., Ji X.Y., Liu C., Yadav P.K., Wu R., Yang P., Liu Z. (2013). IL-25 downregulates Th1/Th17 immune response in an IL-10–dependent manner in inflammatory bowel disease. Inflamm. Bowel Dis..

[69] Patel A.A., Ginhoux F., Yona S. (2021). Monocytes, macrophages, dendritic cells and neutrophils: An update on lifespan kinetics in health and disease. Immunology.

[70] Yip J.L., Balasuriya G.K., Spencer S.J., Hill-Yardin E.L. (2021). The role of intestinal macrophages in gastrointestinal homeostasis: Heterogeneity and implications in disease. Cell. Mol. Gastroenterol. Hepatol..

[71] Viola M.F., Boeckxstaens G. (2020). Guy. Intestinal resident macrophages: Multitaskers of the gut. Neurogastroenterol. Motil..

[72] Rolot M., Dewals B.G. (2018). Macrophage Activation and Functions during Helminth Infection: Recent Advances from the Laboratory Mouse. J. Immunol. Res..

[73] Esser-von Bieren J., Mosconi I., Guiet R., Piersgilli A., Volpe B., Chen F., Gause W.C., Seitz A., Verbeek J.S., Harris N.L. (2013). Antibodies trap tissue migrating helminth larvae and prevent tissue damage by driving IL-4Rα-independent alternative differentiation of macrophages. PLoS Pathog..

[74] Zhao A., Urban J.F., Anthony R.M., Sun R., Stiltz J., Van Rooijen N., Wynn T.A., Shea-Donohue T. (2008). Th2 cytokine-induced alterations in intestinal smooth muscle function depend on alternatively activated macrophages. Gastroenterology.

[75] Coakley G., Harris N.L. (2020). Interactions between macrophages and helminths. Parasite Immunol..

[76] Zhao J., Lv Z., Wang F., Wei J., Zhang Q., Li S., Yang F., Zeng X., Wu X., Wu Z. (2013). Ym1, an eosinophilic chemotactic factor, participates in the brain inflammation induced by Angiostrongylus cantonensis in mice. Parasitol. Res..

[77] Sun Y.J., Chang NC A., Hung S.I., Chang A.C., Chou C.C., Hsiao C.D. (2001). The crystal structure of a novel mammalian lectin, Ym1, suggests a saccharide binding site. J. Biol. Chem..

[78] Nair M.G., Du Y., Perrigoue J.G., Zaph C., Taylor J.J., Goldschmidt M., Swain G.P., Yancopoulos G.D., Valenzuela D.M., Murphy A. (2009). Alternatively activated macrophage-derived RELM-α is a negative regulator of type 2 inflammation in the lung. J. Exp. Med..

[79] Gause W.C., Wynn T.A., Allen J.E. (2013). Type 2 immunity and wound healing: Evolutionary refinement of adaptive immunity by helminths. Nat. Rev. Immunol..

[80] Redpath S.A., Fonseca N.M., Perona-Wright G. (2014). Protection and pathology during parasite infection: IL-10 strikes the balance. Parasite Immunol..

[81] Uciechowski P., Rink L. (2018). Neutrophil, basophil, and eosinophil granulocyte functions in the elderly. Handbook of Immunosenescence.

[82] Mitre E., Nutman T.B. (2003). Lack of basophilia in human parasitic infections. Am. J. Trop. Med. Hyg..

[83] Siracusa M.C., Saenz S.A., Hill D.A., Kim B.S., Headley M.B., Doering T.A., Wherry E.J., Jessup H.K., Siegel L.A., Kambayashi T. (2011). TSLP promotes interleukin-3-independent basophil haematopoiesis and type 2 inflammation. Nature.

[84] Salter B.M., Oliveria J.P., Nusca G., Smith S.G., Tworek D., Mitchell P.D., Watson R.M., Sehmi R., Gauvreau G.M. (2016). IL-25 and IL-33 induce Type 2 inflammation in basophils from subjects with allergic asthma. Respir. Res..

[85] Siracusa M.C., Comeau M.R., Artis D. (2011). New insights into basophil biology: Initiators, regulators, and effectors of type 2 inflammation. Ann. N. Y. Acad. Sci..

[86] Bieneman A.P., Chichester K.L., Chen Y.H., Schroeder J.T. (2005). Toll-like receptor 2 ligands activate human basophils for both IgE-dependent and IgE-independent secretion. J. Allergy Clin. Immunol..

[87] Inclan-Rico J.M., Siracusa M.C. (2018). First responders: Innate immunity to helminths. Trends Parasitol..

[88] Voskamp A.L., Prickett S.R., Mackay F., Rolland J.M., O’Hehir R.E. (2013). MHC class II expression in human basophils: Induction and lack of functional significance. PLoS ONE.

[89] Inclan-Rico J.M., Ponessa J.J., Valero-Pacheco N., Hernandez C.M., Sy C.B., Lemenze A.D., Beaulieu A.M., Siracusa M.C. (2020). Basophils prime group 2 innate lymphoid cells for neuropeptide-mediated inhibition. Nat. Immunol..

[90] Kim H.J., Jung Y. (2020). The emerging role of eosinophils as multifunctional leukocytes in health and disease. Immune Netw..

[91] Park Y.M., Bochner B.S. (2010). Eosinophil survival and apoptosis in health and disease. Allergy Asthma Immunol. Res..

[92] Wen T., Besse J.A., Mingler M.K., Fulkerson P.C., Rothenberg M.E. (2013). Eosinophil adoptive transfer system to directly evaluate pulmonary eosinophil trafficking in vivo. Proc. Natl. Acad. Sci. USA.

[93] Lee J.J., Jacobsen E.A., Ochkur S.I., McGarry M.P., Condjella R.M., Doyle A.D., Luo H., Zellner K.R., Protheroe C.A., Willetts L. (2012). Human versus mouse eosinophils: “That which we call an eosinophil, by any other name would stain as red”. J. Allergy Clin. Immunol..

[94] Rosenberg H.F., Dyer K.D., Foster P.S. (2013). Eosinophils: Changing perspectives in health and disease. Nat. Rev. Immunol..

[95] Kvarnhammar A.M., Cardell L.O. (2012). Pattern-recognition receptors in human eosinophils. Immunology.

[96] Månsson A., Cardell L.-O. (2009). Role of atopic status in Toll-like receptor (TLR) 7-and TLR9-mediated activation of human eosinophils. J. Leucoc. Biol..

[97] Acharya K.R., Ackerman S.J. (2014). Eosinophil granule proteins: Form and function. J. Biol. Chem..

[98] Shamri R., Xenakis J.J., Spencer L.A. (2011). Spencer. Eosinophils in innate immunity: An evolving story. Cell Tissue Res..

[99] Meeusen E., Balic A. (2000). Do eosinophils have a role in the killing of helminth parasites?. Parasitol. Today.

[100] Huang L., Appleton J.A. (2016). Eosinophils in helminth infection: Defenders and dupes. Trends Parasitol..

[101] Summers C., Rankin S.M., Condliffe A.M., Singh N., Peters A.M., Chilvers E.R. (2010). Neutrophil kinetics in health and disease. Trends Immunol..

[102] Pesce J.T., Liu Z., Hamed H., Alem F., Whitmire J., Lin H., Liu Q., Urban J.F., Gause W.C. (2008). Neutrophils clear bacteria associated with parasitic nematodes augmenting the development of an effective Th2-type response. J. Immunol..

[103] Gayen P., Maitra S., Datta S., Sinha Babu S.P. (2010). Evidence for Wolbachia symbiosis in microfilariae of Wuchereria bancrofti from West Bengal, India. J. Biosci..

[104] Murfin K.E., Dillman A.R., Foster J.M., Bulgheresi S., Slatko B.E., Sternberg P.W., Goodrich-Blair H. (2012). Nematode-bacterium symbioses—Cooperation and conflict revealed in the “Omics” age. Biol. Bull..

[105] Buys J., Wever R., Ruitenberg E.J. (1984). Myeloperoxidase is more efficient than eosinophil peroxidase in the in vitro killing of newborn larvae of Trichinella spiralis. Immunology.

[106] Bonne-Année S., Kerepesi L.A., Hess J.A., Wesolowski J., Paumet F., Lok J.B., Nolan T.J., Abraham D. (2014). Extracellular traps are associated with human and mouse neutrophil and macrophage mediated killing of larval Strongyloides stercoralis. Microbes Infect..

[107] Heeb L.E., Egholm C., Impellizzieri D., Ridder F., Boyman O. (2018). Regulation of neutrophils in type 2 immune responses. Curr. Opin. Immunol..

[108] Sutherland T.E., Logan N., Rückerl D., Humbles A.A., Allan S.M., Papayannopoulos V., Stockinger B., Maizels R.M., Allen J.E. (2014). Chitinase-like proteins promote IL-17-mediated neutrophilia in a tradeoff between nematode killing and host damage. Nat. Immunol..

[109] Penttila I.A., Ey P.L., Jenkin C.R. (1984). Infection of mice with Nematospiroides dubius: Demonstration of neutrophil-mediated immunity in vivo in the presence of antibodies. Immunology.

[110] Middleton D., Garza J.J., Greiner S.P., Bowdridge S.A. (2020). Neutrophils rapidly produce Th2 cytokines in response to larval but not adult helminth antigen. Parasite Immunol..

[111] Moro K., Koyasu S. (2010). Innate production of Th2 cytokines by adipose tissue-associated c-Kit+ Sca-1+ lymphoid cells (89.11). J. Immunol..

[112] Kobayashi T., Motomura Y., Moro K. (2021). The discovery of group 2 innate lymphoid cells has changed the concept of type 2 immune diseases. Int. Immunol..

[113] Herbert D.B., Douglas B., Zullo K. (2019). Group 2 innate lymphoid cells (ILC2): Type 2 immunity and helminth immunity. Int. J. Mol. Sci..

[114] Nausch N., Mutapi F. (2018). Group 2 ILCs: A way of enhancing immune protection against human helminths?. Parasite Immunol..

[115] Neill D.R., Wong S.H., Bellosi A., Flynn R.J., Daly M., Langford T.K., Bucks C., Kane C.M., Fallon P.G., Pannell R. (2010). Nuocytes represent a new innate effector leukocyte that mediates type-2 immunity. Nature.

[116] Oliphant C.J., Hwang Y.Y., Walker J.A., Salimi M., Wong S.H., Brewer J.M., Englezakis A., Barlow J.L., Hams E., Scanlon S.T. (2014). MHCII-mediated dialog between group 2 innate lymphoid cells and CD4+ T cells potentiates type 2 immunity and promotes parasitic helminth expulsion. Immunity.

[117] Ghably J., Saleh H., Vyas H., Peiris E., Misra N., Krishnaswamy G. (2015). Paul Ehrlich’s mastzellen: A historical perspective of relevant developments in mast cell biology. Mast Cells: Methods and Protocols.

[118] da Silva E.Z., Jamur M.C., Oliver C. (2014). Mast cell function: A new vision of an old cell. J. Histochem. Cytochem..

[119] Ryan N.M., Oghumu S. (2019). Role of mast cells in the generation of a T-helper type 2 dominated anti-helminthic immune response. Biosci. Rep..

[120] Wernersson S., Pejler G. (2014). Mast cell secretory granules: Armed for battle. Nat. Rev. Immunol..

[121] Paivandy A., Pejler G. (2021). Novel strategies to target mast cells in disease. J. Innate Immun..

[122] Galli S.J., Borregaard N., Wynn T.A. (2011). Phenotypic and functional plasticity of cells of innate immunity: Macrophages, mast cells and neutrophils. Nat. Immunol..

[123] Simon T., László V., Falus A. (2011). Impact of histamine on dendritic cell functions. Cell Biol. Int..

[124] Patente T.A., Pinho M.P., Oliveira A.A., Evangelista G.C.M., Bergami-Santos P.C., Barbuto J.A.M. (2019). Human dendritic cells: Their heterogeneity and clinical application potential in cancer immunotherapy. Front. Immunol..

[125] Poulsen L.K., Hummelshoj L. (2007). Triggers of IgE class switching and allergy development. Ann. Med..

[126] Nutman T.B. (2015). Looking beyond the induction of Th2 responses to explain immunomodulation by helminths. Parasite Immunol..

[127] Zheng W.P., Flavell R.A. (1997). The transcription factor GATA-3 is necessary and sufficient for Th2 cytokine gene expression in CD4 T cells. Cell.

[128] Zhu J., Cote-Sierra J., Guo L., Paul W.E. (2003). Stat5 activation plays a critical role in Th2 differentiation. Immunity.

[129] Bruns H.A., Schindler U., Kaplan M.H. (2003). Expression of a constitutively active Stat6 in vivo alters lymphocyte homeostasis with distinct effects in T and B cells. J. Immunol..

[130] Zhu J., Min B., Hu-Li J., Watson C.J., Grinberg A., Wang Q., Killeen N., Urban J.F., Guo L., Paul W.E. (2004). Conditional deletion of Gata3 shows its essential function in TH1-TH2 responses. Nat. Immunol..

[131] Zhu J., Yamane H., Paul W.E. (2009). Differentiation of effector CD4 T cell populations. Annu. Rev. Immunol..

[132] Pereira J.P., Kelly L.M., Xu Y., Cyster J.G. (2009). EBI2 mediates B cell segregation between the outer and centre follicle. Nature.

[133] Savage P.A., Klawon D.E., Miller C.H. (2020). Regulatory T cell development. Annu. Rev. Immunol..

[134] Zheng Y., Wang Z., Deng L., Zhang G., Yuan X., Huang L., Xu W., Shen L. (2015). Modulation of STAT3 and STAT5 activity rectifies the imbalance of Th17 and Treg cells in patients with acute coronary syndrome. Clin. Immunol..

[135] Schmitt E.G., Williams C.B. (2013). Generation and function of induced regulatory T cells. Front. Immunol..

[136] Satoguina J.S., Adjobimey T., Arndts K., Hoch J., Oldenburg J., Layland L.E., Hoerauf A. (2008). Tr1 and naturally occurring regulatory T cells induce IgG4 in B cells through GITR/GITR-L interaction, IL-10 and TGF-β. Eur. J. Immunol..

[137] Van Der Neut Kolfschoten M., Schuurman J., Losen M., Bleeker W.K., Martínez- Martínez P., Vermeulen E., Den Bleker T.H., Wiegman L., Vink T., Aarden L.A. (2007). Anti-inflammatory activity of human IgG4 antibodies by dynamic Fab arm exchange. Science.

[138] McSorley H.J., Maizels R.M. (2012). Helminth infections and host immune regulation. Clin. Microbiol. Rev..

[139] Johnston C.J., Smyth D.J., Kodali R.B., White M.P., Harcus Y., Filbey K.J., Hewitson J.P., Hinck C.S., Ivens A., Kemter A.M. (2017). A structurally distinct TGF-β mimic from an intestinal helminth parasite potently induces regulatory T cells. Nat. Commun..

[140] White M.P., McManus C.M., Maizels R.M. (2020). Regulatory T-cells in helminth infection: Induction, function and therapeutic potential. Immunology.

[141] Cable J., Harris P.D., Lewis J.W., Behnke J.M. (2006). Molecular evidence that *Heligmosomoides polygyrus* from laboratory mice and wood mice are separate species. Parasitology.

[142] El Ridi R., Ozaki T., Kamiya H. (1998). *Schistosoma mansoni* infection in IgE-producing and IgE-deficient mice. J. Parasitol..

[143] McCoy K.D., Stoel M., Stettler R., Merky P., Fink K., Senn B.M., Schaer C., Massacand J., Odermatt B., Oettgen H.C. (2008). Polyclonal and specific antibodies mediate protective immunity against enteric helminth infection. Cell Host Microbe.

[144] McVay C.S., Bracken P., Gagliardo L.F., Appleton J. (2000). Antibodies to tyvelose exhibit multiple modes of interference with the epithelial niche of Trichinella spiralis. Infect. Immun..

[145] Harris N., Gause W.C. (2011). To B or not to B: B cells and the Th2-type immune response to helminths. Trends Immunol..

[146] Nimmerjahn F., Ravetch J.V. (2011). FcγRs in health and disease. Negative Co-Receptors and Ligands.

[147] Moulin V., Andris F., Thielemans K., Maliszewski C., Urbain J., Moser M. (2000). B lymphocytes regulate dendritic cell (DC) function in vivo: Increased interleukin 12 production by DCs from B cell–deficient mice results in T helper cell type 1 deviation. J. Exp. Med..

[148] Ferru I., Roye O., Delacre M., Auriault C., Wolowczuk I. (1998). Infection of B-cell-deficient mice by the parasite Schistosoma mansoni: Demonstration of the participation of B cells in granuloma modulation. Scand. J. Immunol..

[149] Kaufmann S.H. (2016). Immunopathology of mycobacterial diseases. Seminars in Immunopathology.

[150] Co D.O., Hogan L.H., Il-Kim S., Sandor M. (2004). T cell contributions to the different phases of granuloma formation. Immunol. Lett..

[151] Malta K.K., Silva T.P., Palazzi C., Neves V.H., Carmo L.A., Cardoso S.J., Melo R.C. (2021). Changing our view of the Schistosoma granuloma to an ecological standpoint. Biol. Rev..

[152] Anthony R.M., Rutitzky L.I., Urban J.F., Stadecker M.J., Gause W.C. (2007). Protective immune mechanisms in helminth infection. Nat. Rev. Immunol..

[153] Kayes S.G., Jones R.E., Omholt P.E. (1988). Pulmonary granuloma formation in murine toxocariasis: Transfer of granulomatous hypersensitivity using bronchoalveolar lavage cells. J. Parasitol..

[154] Kayes S.G., Oaks J.A. (1978). Development of the granulomatous response in murine toxocariasis. Initial events. Am. J. Pathol..

[155] Ta Llanwarne F., Helmby H. (2021). Granuloma formation and tissue pathology in Schistosoma japonicum versus Schistosoma mansoni infections. Parasite Immunol..

[156] Schwartz C., Fallon P.G. (2018). Schistosoma “eggs-iting” the host: Granuloma formation and egg excretion. Front. Immunol..

[157] Stadecker M.J., Asahi H., Finger E., Hernandez H.J., Rutitzky L.I., Sun J. (2004). The immunobiology of Th1 polarization in high-pathology schistosomiasis. Immunol. Rev..

[158] Pagán A.J., Ramakrishnan L. (2018). The formation and function of granulomas. Annu. Rev. Immunol..

[159] Peterson P.K., Gekker G., Hu S., Anderson W.R., Teichert M., Chao C.C., Molitor T.W. (1996). Multinucleated giant cell formation of swine microglia induced by Mycobacterium bovis. J. Infect. Dis..

[160] Lay G., Poquet Y., Salek-Peyron P., Puissegur M.P., Botanch C., Bon H., Levillain F., Duteyrat J.L., Emile J.F., Altare F. (2007). Langhans giant cells from *M. tuberculosis*-induced human granulomas cannot mediate mycobacterial uptake. J. Pathol. A J. Pathol. Soc. Great Br. Irel..

[161] Herrtwich L., Nanda I., Evangelou K., Nikolova T., Horn V., Sagar S., Erny D., Stefanowski J., Rogell L., Klein C. (2016). DNA damage signaling instructs polyploid macrophage fate in granulomas. Cell.

[162] Puissegur M.P., Lay G., Gilleron M., Botella L., Nigou J., Marrakchi H., Mari B., Duteyrat J.L., Guerardel Y., Kremer L. (2007). Mycobacterial lipomannan induces granuloma macrophage fusion via a TLR2-dependent, ADAM9-and β1 integrin-mediated pathway. J. Immunol..

[163] Reynolds L.A., Filbey K.J., Maizels R.M. (2012). Immunity to the model intestinal helminth parasite Heligmosomoides polygyrus. Seminars in Immunopathology.

[164] Amaral K.B., Silva T.P., Dias F.F., Malta K.K., Rosa F.M., Costa-Neto S.F., Gentile R., Melo R.C. (2017). Histological assessment of granulomas in natural and experimental Schistosoma mansoni infections using whole slide imaging. PLoS ONE.

[165] Chiaramonte M.G., Schopf L.R., Neben T.Y., Cheever A.W., Donaldson D.D., Wynn T.A. (1999). IL-13 is a key regulatory cytokine for Th2 cell-mediated pulmonary granuloma formation and IgE responses induced by Schistosoma mansoni eggs. J. Immunol..

[166] Hesse M., Modolell M., La Flamme A.C., Schito M., Fuentes J.M., Cheever A.W., Pearce E.J., Wynn T.A. (2001). Differential regulation of nitric oxide synthase-2 and arginase-1 by type 1/type 2 cytokines in vivo: Granulomatous pathology is shaped by the pattern of L-arginine metabolism. J. Immunol..

[167] Chuah C., Jones M.K., Burke M.L., McManus D.P., Gobert G.N. (2014). Cellular and chemokine-mediated regulation in schistosome-induced hepatic pathology. Trends Parasitol..

[168] Boros D.L. (1989). Immunopathology of Schistosoma mansoni infection. Clin. Microbiol. Rev..

[169] Gryseels B., Polman K., Clerinx J., Kestens L. (2006). Human schistosomiasis. Lancet.

[170] Wilson M.S., Mentink-Kane M.M., Pesce J.T., Ramalingam T.R., Thompson R., Wynn T.A. (2007). Immunopathology of schistosomiasis. Immunol. Cell Biol..

[171] Dunne D.W., Hassounah O., Musallam R., Lucas S., Pepys M.B., Baltz M., Doenhoff M. (1983). Mechanisms of Schistosoma mansoni egg excretion: Parasitological observations in immunosuppressed mice reconstituted with immune serum. Parasite Immunol..

[172] Giorgio S., Gallo-Francisco P.H., Roque G.A.S., Floro e Silva M. (2020). Granulomas in parasitic diseases: The good and the bad. Parasitol. Res..

[173] Vuitton D.A. (2003). The ambiguous role of immunity in echinococcosis: Protection of the host or of the parasite?. Acta Trop..

[174] Brunet L.R. (2001). Nitric oxide in parasitic infections. Int. Immunopharmacol..

[175] Takaki K.K., Rinaldi G., Berriman M., Pagán A.J., Ramakrishnan L. (2021). Schistosoma mansoni eggs modulate the timing of granuloma formation to promote transmission. Cell Host Microbe.

